# Dataset concerning GroEL chaperonin interaction with proteins

**DOI:** 10.1016/j.dib.2016.01.008

**Published:** 2016-01-13

**Authors:** V.V. Marchenkov, N.Yu. Marchenko, A.L. Kaysheva, N.V. Kotova, I.A. Kashparov, G.V. Semisotnov

**Affiliations:** Institute of Protein Research, RAS, Russia

## Abstract

GroEL chaperonin is well-known to interact with a wide variety of polypeptide chains. Here we show the data related to our previous work (http://dx.doi.org/10.1016/j.pep.2015.11.020[1]), and concerning the interaction of GroEL with native (lysozyme, α-lactalbumin) and denatured (lysozyme, α-lactalbumin and pepsin) proteins in solution. The use of affinity chromatography on the base of denatured pepsin for GroEL purification from fluorescent impurities is represented as well.

## Specifications table

**Table:** 

Subject area	*Biology*
More specific subject area	*Protein–protein interaction*
Type of data	*Figures*
How data was acquired	*Fluorescence spectroscopy data was acquired using a RF-5301PC spectrofluorometer (Shimadzu, Japan), while affinity chromatography was performed using a Minipuls 3 peristaltic pump (Gilson, France) and registration system Uvicord S 2138 (LKB, Sweden)*
Data format	*Analyzed*
Experimental factors	*Protein labelling with fluorescein isothiocyanate. Covalent immobilization of pepsin to BrCN-Sepharose.*
Experimental features	*Titration was obtained by stock solutions in a standard quartz cuvette 1x1×4* *cm*^3^*at intensive mixing. Affinity chromatography was performed using 2* *ml sorbent placed in a small column equilibrated with the loading buffer.*
Data source location	*Pushchino, Moscow Region, Russia*
Data accessibility	*Data is within this article*

## Value of the data

• Understanding of GroEL functioning.
• Purification of GroEL from impurities.
• Separation of GroEL and recombinant proteins of interest after co-expression of their genes.
• Evaluation of apparent dissociation constants of the complexes.

## 1 Data

**Fig. 1:**
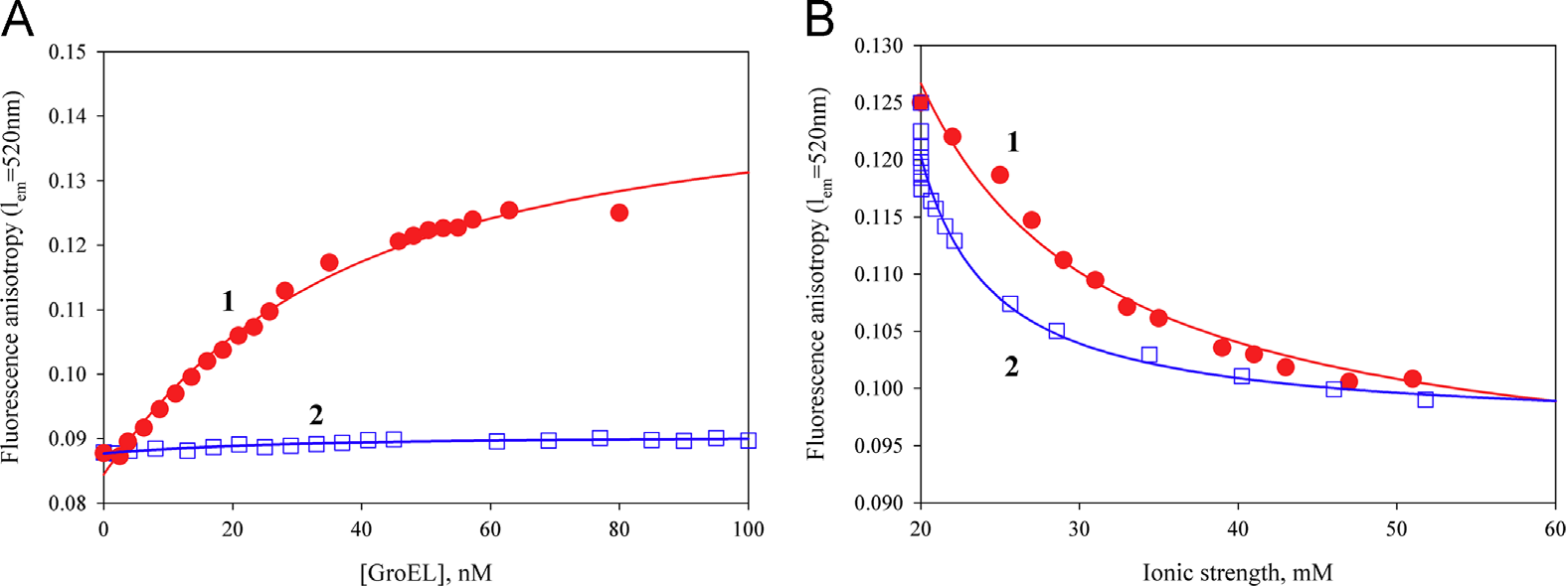
(A) Titration of native fluorescein-labeled lysozyme (1) and human α-lactalbumin (2) with GroEL at a low ionic strength (20 mM Tris–HCl, pH 7.5), and (B) titration of native lysozyme with NaCl (1) and MgCl_2_ (2) in the presence of 80 nM GroEL. The concentration of fluorescein-labeled proteins is 30×10^−9^ M. The apparent dissociation constant (*K*_*diss*_^*app*^) evaluated from approximation of native lysozyme titration curve (solid line) is 50×10^−9^ M.

**Fig. 2:**
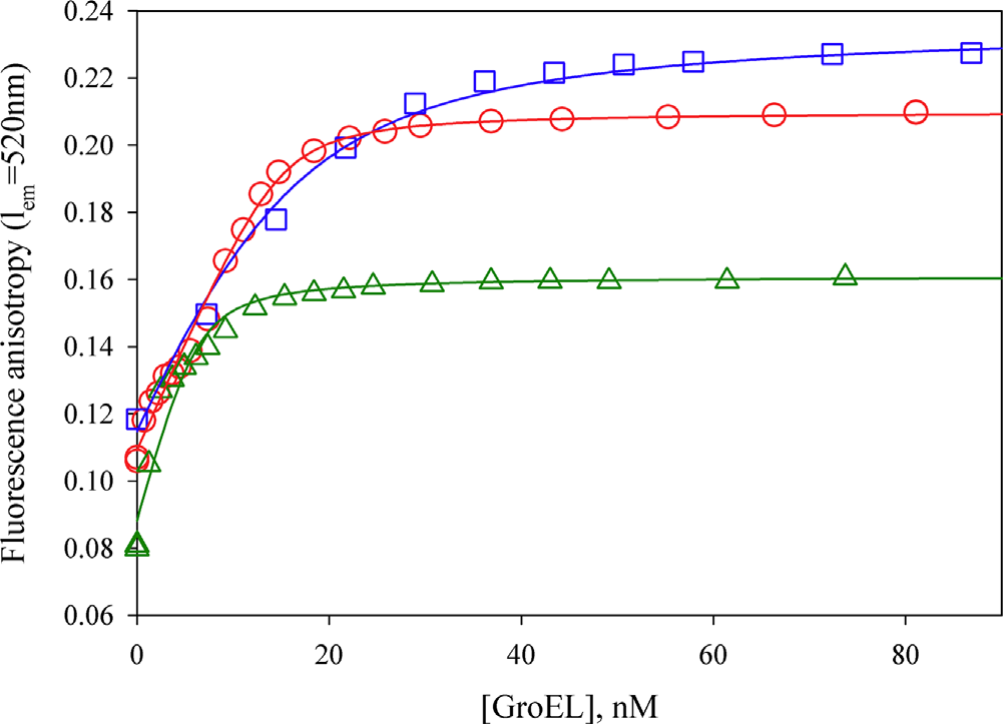
Dependence of fluorescence anisotropy of fluorescein-labeled denatured lysozyme (-o-) and human α-lactalbumin (-∆-) in 20 mM Tris–HCl, pH 7.5, 20 mM DTT, 10 mM MgCl_2_, 300 mM NaCl as well as of pepsin (-□-) in 20 mM Tris–HCl, pH 7.5, 10 mM MgCl_2_, 600 mM NaCl. The protein concentration is 30×10^−9^ M. Apparent dissociation constants (*K*_*diss*_^*app*^) evaluated from approximation of titration curves (solid lines) are 1.4×10^−9^ M for denatured lysozyme, 9.7×10^−9^ M for denatured pepsin, and 1.6×10^−9^ M for denatured α-lactalbumin.

**Fig. 3:**
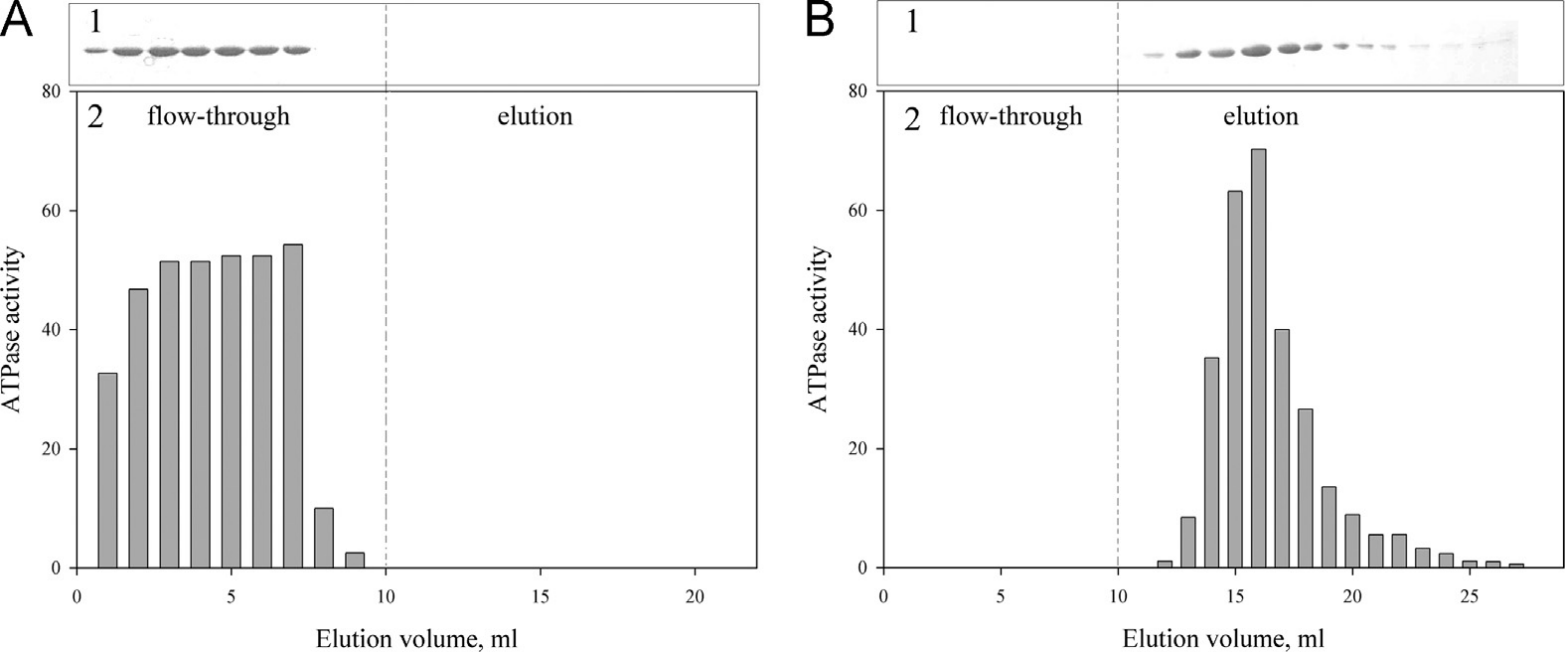
Affinity chromatography of GroEL (previously purified by a standard technique [2], [3]) using an affinity sorbent on the base of denatured pepsin. (A) Loading in 20 mM Tris–HCl, pH 7.5; elution with 20 mM Tris–HCl, pH 7.5, 8 M urea. (B) Loading in 20 mM Tris–HCl, pH 7.5, 300 mM NaCl, 10 mM MgCl_2_; elution with 20 mM Tris–HCl, pH 7.5. Chromatographic profiles are obtained by SDS gel electrophoresis (1) and by measurements of GroEL ATPase activity (2).

**Fig. 4:**
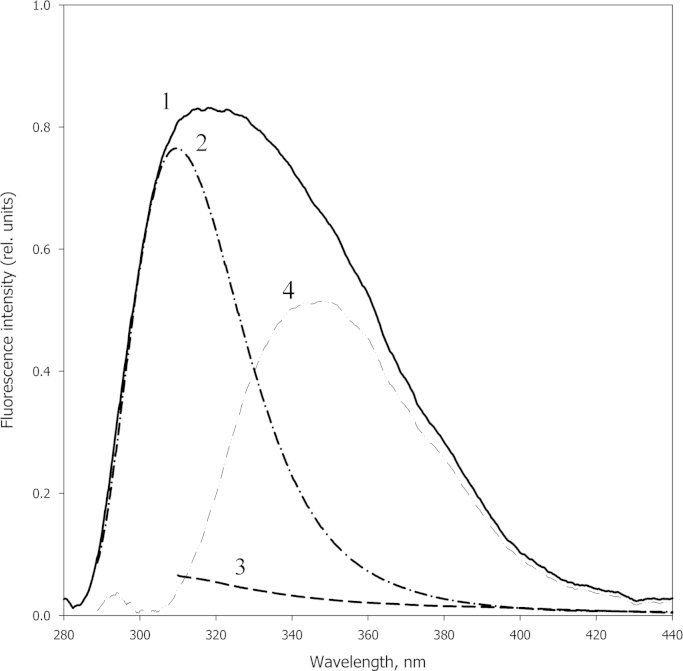
Fluorescence spectra of GroEL (*C*=0.1 mg/ml) before (1) and after (2,3) affinity chromatography on the base of denatured pepsin (see Fig. 3). Buffer: 20 mM Tris–HCl, pH 7.5. Excitation wavelengths are 270 nm (1,2) and 293 nm (3). (4) The difference spectrum (1−2).

Using polarized fluorescence, it is shown that GroEL is able to interact with the native conformational state of proteins (in particular, lysozyme) when the ionic strength is low (~20 mM) and the protein is positively charged (Fig. 1). The GroEL interaction with denatured proteins is also dependent on the ionic strength of solution (Fig. 2, Fig. 3). Affinity chromatography on the base of denatured pepsin allows purification of GroEL from impurities tightly bound with it after standard technique of GroEL isolation from cells (Fig. 3, Fig. 4).

## 2 Experimental design, materials and methods

GroEL was purified from *Escherichia coli* cells (HB101 strain) after expression of the multicopy plasmid pGroE4 according to the standard technique [2], [3]. Chicken egg white lysozyme, pepsin from porcine gastric mucosa and human α-lactalbumin were purchased from “Sigma” (USA) and used without additional purification. Fluorescein-labeled proteins were prepared by incubation of protein solution (10 mg/ml) in 2 ml 0.15 M KNaCO_3_ (pH 9.1) in the presence of 2 mM fluorescein isothiocyanate during 5 h at 20 °C. The reaction mixture was desalted using a PD-10 column equilibrated with 25 mM L-His buffer (pH 5.85). Additional purification and separation of the protein molecules containing different amounts of fluorescein labels were performed using a MonoQ HR 5/5 column equilibrated with 2 mM L-His buffer (pH 5.85) and elution gradient 0–1 M NaCl.

The protein concentration was measured spectrophotometrically using extinction at 280 nm (OD of 1 mg/ml and 1 cm optical length): 1.6 for human α-lactalbumin, 2.7 for lysozyme, 1.4 for pepsin, 0.25 and 0.19 for GroEL before and after additional purification from impurities.

GroEL ATPase activity was measured spectrophotometrically using malachite green hydrochloride according to the published protocol [4].

### 2.1 Titration of fluorescein-labeled proteins with a GroEL excess allows evaluating their affinity to the chaperonin in solution

To choose the proteins for making affinity sorbents [1], we evaluated the apparent dissociation constants of GroEL complexes with a number of proteins using titration experiments (Fig. 1, Fig. 2).

Fluorescence anisotropy of fluorescein-labeled proteins was measured with a spectrofluorometer RF-5301PC (Shimadzu, Japan) at 490 nm excitation (slit of 3 nm) and 520 nm emission (slit of 10 nm). The change in the fluorescein anisotropy value reflects the change in rotational mobility of the fluorescent label bound with a relatively small protein molecule (molecular weight ~15 kDa), when it interacts or dissociates from a large GroEL molecule (molecular weight ~800 kDa).

The apparent dissociation constants of the GroEL complex with substrate proteins were evaluated from approximation of the titration data using the following equation:

$$f=\{A_{f}/P_{0}\times {\left[P_{0}–X–{K_{diss}}^{app}+{\left(P_{0}+X+{K_{diss}}^{app}\right)}^{2}–4\times X\times P_{0}\right]}^{1/2}+\left(A_{b}/P_{0}\right)\times [P_{0}–{\left(P_{0}–X–K_{diss}+{\left({P_{0}}^{2}+X^{2}+K_{diss}\right)}^{2}–4\times X\times P_{0}\right]}^{1/2}\}.$$

The approximation function was calculated from simple equations

$${K_{diss}}^{app}=\frac{P\times G}{PG},P_{0}=P+PG\;\mathrm{and}\;X=G+PG,$$

where *P* is the free (unbound) substrate protein concentration, *P*_*0*_ is the total substrate protein concentration, *G* is the concentration of free GroEL cavities, *X* is the concentration of all GroEL cavities (i. e. the doubled GroEL concentration), *PG* is the concentration of the complex of GroEL with the substrate protein, *A*_*f*_ and *A*_*b*_ are anisotropy values of free and bound substrate proteins, respectively, and *K*_*diss*_^*app*^ is the apparent dissociation constant.

Fig. 1 A represents titration of fluorescein-labeled native lysozyme and human α-lactalbumin with GroEL at a low ionic strength. As seen, anisotropy of native α-lactalbumin does not change at an increase in the GroEL concentration, while anisotropy of native lysozyme increases reflecting the interaction of this protein with GroEL. This interaction (with the apparent dissociation constant 50×10^−9^ M) is destroyed at addition of NaCl or MgCl_2_ (Fig. 1B). When the proteins are denatured, their affinity to GroEL considerably increases to nanomolar dissociation constants (Fig. 2). Note that negatively charged denatured proteins (human α-lactalbumin and pepsin) do not interact with GroEL at a low ionic strength and in the absence of divalent ions, while they strongly interact with GroEL at a high ionic strength or in the presence of divalent ions [5], [6], [7]. Affinity chromatography reveals the same events as in solution [5]. This peculiarity allows purifying GroEL from impurities tightly bound to GroEL during its isolation from cell [8], [9].

### 2.2 Purification of isolated GroEL from impurities using a denatured pepsin affinity sorbent

Fig. 3 represents profiles of GroEL affinity chromatography on the base of denatured pepsin obtained by SDS gel electrophoresis and by measurements of GroEL ATPase activity. The data show that at a low ionic strength (20 mM Tris–HCl), pepsin (denatured at neutral pH [10]) does not interact with GroEL and all chaperonin is collected as flow-through (Fig. 3A). At a high ionic strength (~300 mM NaCl and 10 mM MgCl_2_), denatured pepsin tightly interacts with GroEL, and is washed off with a low ionic strength buffer. GroEL affinity to denatured pepsin seems to be stronger than to tryptophan-containing impurities [9], because the tryptophan fluorescence component disappears from the GroEL fluorescence spectrum after affinity chromatography (Fig. 4).
